# Women’s Preferences for Treatment of Perinatal Depression and Anxiety: A Discrete Choice Experiment

**DOI:** 10.1371/journal.pone.0156629

**Published:** 2016-06-03

**Authors:** Jemimah Ride, Emily Lancsar

**Affiliations:** Centre for Health Economics, Monash University, Melbourne, Australia; St Francis Hospital, UNITED STATES

## Abstract

Perinatal depression and anxiety (PNDA) are an international healthcare priority, associated with significant short- and long-term problems for women, their children and families. Effective treatment is available but uptake is suboptimal: some women go untreated whilst others choose treatments without strong evidence of efficacy. Better understanding of women’s preferences for treatment is needed to facilitate uptake of effective treatment. To address this issue, a discrete choice experiment (DCE) was administered to 217 pregnant or postnatal women in Australia, who were recruited through an online research company and had similar sociodemographic characteristics to Australian data for perinatal women. The DCE investigated preferences regarding cost, treatment type, availability of childcare, modality and efficacy. Data were analysed using logit-based models accounting for preference and scale heterogeneity. Predicted probability analysis was used to explore relative attribute importance and policy change scenarios, including how these differed by women’s sociodemographic characteristics. Cost and treatment type had the greatest impact on choice, such that a policy of subsidising effective treatments was predicted to double their uptake compared with the base case. There were differences in predicted uptake associated with certain sociodemographic characteristics: for example, women with higher educational attainment were more likely to choose effective treatment. The findings suggest policy directions for decision makers whose goal is to reduce the burden of PNDA on women, their children and families.

## 1. Introduction

The perinatal period (pregnancy until 12 months after the child’s birth) is a critical stage in a woman’s life. Depression and anxiety during this time are associated with significant burden on women and their families [1], and with increased health care costs [2–4]. Perinatal depression is common across countries and cultures [5, 6]; prevalence estimates vary but are usually accepted to be between 10–20% [7, 8]. Perinatal depression and anxiety (PNDA) are often comorbid [9, 10]. Although the symptoms often resolve within the first six months, for many women they are ongoing one or two years after the baby’s birth [11].

This burden extends beyond the perinatal period: women who have experienced perinatal depression are more likely to have recurrent or chronic depression [1, 10, 12–14]. It also extends beyond the mother: maternal mental health problems are associated with problems in the maternal-infant relationship [1], with psychological, behavioural, cognitive and health problems in children [15–17], and with difficulties in the intimate partner relationship [1].

Treatments for PNDA recommended by evidence-based guidelines include individual- or group-based psychological therapies (such as cognitive-behavioural and interpersonal therapies), medication (particularly antidepressants) and psychosocial interventions (e.g. peer support) [18–21]. Such treatments have been shown to reduce symptoms as well as improve the chance of recovery within the first postnatal year [20]. The evidence for these interventions largely derives from high-income countries, but there is some research suggesting similar interventions are effective in low-and middle-income countries, albeit with the need for contextual adaptation [22–24]. The different types of treatment have differing implications for the allocation of finite resources in perinatal mental healthcare (e.g. costs, provider type, and duration). In Australia, the setting for this study, psychological therapies, medication and psychosocial interventions are funded through a mixture of public and private health insurance and out-of-pocket patient costs.

However, not all women with PNDA receive such treatment. Some go undiagnosed, which may reflect under-recognition and stigmatisation of PNDA by women and healthcare practitioners. The natural inference is that screening might be of value in order to improve identification of women with or at risk of PNDA, but there is longstanding debate over the clinical- and cost-effectiveness of screening in this context (for a recent example, see [25, 26]). Highlighted in this debate is the issue of managing women after the process of identification. Even among those recognised to have symptoms, the treatment rate is approximately 60% in high-income countries [1, 27]. Identified barriers to treatment include perceived stigma, time pressures, cost, childcare difficulties, limited service availability and concerns about taking medication whilst pregnant or breastfeeding [28–31]. Some women refuse treatment, and others take up treatments without strong evidence of efficacy, such as acupuncture, massage, traditional Chinese medicine, homeopathy and herbal therapy [32, 33]. Postulated facilitators include at-home treatment provision, training perinatal care providers, educating women about PNDA and its treatment, and streamlining referral processes [29]. Barriers to access and lack of service availability may be particularly important for those in low- or middle-income countries [34, 35] and those from ethnic minorities or low-income groups in high-income countries [36].

Increasing the effective treatment of PNDA, by reducing both non-treatment and the use of treatments without sound evidence of efficacy, would capitalise on the opportunity to improve women’s perinatal mental health, and so reduce the burden on women, their families and health services [37]. However, to be effective, an intervention must be not only efficacious in trials, but also available to and utilised by consumers. Understanding the gap between the identification and implementation of efficacious treatments is a research priority that can facilitate policy responses and service design to increase effective treatment [38]. Aligning treatment to patient preferences is a recognised objective in perinatal mental healthcare [18] since it may improve women’s acceptance of treatment and their treatment outcomes [39, 40], provided that preferred treatment services are available.

However, it is not known to what degree the nature of treatment packages affects their uptake. Many studies of women’s preferences for treatment of PNDA do not quantify the impact of each barrier or facilitator. Most also focus on recommended treatments and those in the public health system, not exploring those that women might see as viable alternatives, such as complementary, alternative and allied health interventions.

The aim of this study is to explore women’s preferences for the attributes of PNDA treatment packages and their impact on initiation of treatment. Although other factors than patient preference contribute to treatment choice, including individual- and structural-level factors such as relationship with providers and service availability, insight into women’s preferences for treatment of PNDA can assist decision makers in the design and provision of services to optimise uptake of effective treatment.

A discrete choice experiment (DCE) was chosen to explore women’s preferences for two reasons. First, observational (or revealed preference) data in this area are limited. Such studies lack detail both of the attributes of a chosen treatment package and of the non-selected alternatives available to each individual. A DCE overcomes this problem by creating a hypothetical market in which the attributes of all alternatives are known. Secondly, a DCE allows evaluation of hypothetical treatment packages, with attribute combinations that may not yet be available [41], a feature particularly important for this study.

DCEs are commonly used for the elicitation of health care preferences [42]. Applications in mental health include treatment types [43], tests for depression [44] and treatment modalities [45, 46]. One examined public willingness-to-pay for counselling regarding antidepressant use in pregnancy [47], another explored effects of mental health problems on obstetric care preferences [48]. However, to our knowledge this is the first DCE examining women’s preferences for PNDA treatment.

DCEs elicit preferences for the attributes (characteristics) of a good or service by creating a hypothetical market, such as a range of PNDA treatment services, among which participants make choices. DCEs premise that people’s choices are based on the benefit (utility) they expect to derive from a service, an expectation arising from their preferences for its attributes, rather than for the service as a whole [49]. Participants are presented with a series of choice sets in which they are asked to choose between alternatives, each described as a package of attributes. By systematic construction of the choice sets and analysis of participants’ choices, we can quantify the relative importance of the attributes to participants’ choices, and evaluate policies involving changes to these attributes in terms of predicted uptake. Such quantitative prediction of policy impact is a key contribution of this research.

Our objectives in conducting this DCE were: to identify the relevant attributes of treatment for inclusion; to develop a survey tool that gave participants an understanding of the context and set up the hypothetical choice scenario; to conduct the study among women whose preferences were relevant to the research question; to estimate an econometric model that allowed for heterogeneity and fitted the data well; and to interpret the results, including policy-relevant predictions, applying the findings in ways that are meaningful and useful to decision makers. In this paper, we first present the methods employed, including identification of appropriate attributes, design of the choice sets offered to participants, and an overview of the econometric analyses applied to the data. We then present the resulting estimation of the attributes’ relative importance to women’s preferences, explore the policy implications for increasing effective treatment, and discuss ways this research might be extended.

## 2. Methods

Each choice set comprised two unlabelled alternatives (“Treatment A” and “Treatment B”) and a “No Treatment” alternative, with participants required to choose one of these three. Employing unlabelled alternatives allowed us to explore combinations of the attributes rather than defining each alternative by the treatment type. The inclusion of the no-treatment alternative, rather than a forced choice between two treatment alternatives, was essential to explore women’s reasons for choosing treatment over non-treatment.

### 2.1 Development of attributes and levels

Table 1 summarises the five attributes explored in this study, which were derived through review of the literature, discussion with experts (three clinicians and researchers in perinatal mental health), and two focus groups with potential consumers (10 women of childbearing age). The attributes and survey were further refined through preliminary testing of the survey with a further seven potential consumers, in which they were interviewed using a series of open- and closed-ended questions after completion of the survey. (Full attribute descriptions from the final survey are presented in S1 Text.

**Table 1:** Attributes and levels.

Attribute	Levels
Treatment type	Individual counselling^+^
	Combination of counselling and medication^+^
	Group counselling^+^
	Peer support^+^
	Natural, herbal or traditional Chinese medicine
	Meditation, yoga or exercise
	Early parenting centre programme
	Medication*^+^
Cost per session	$0*
	$5
	$50
	$200
Chance of improving symptoms	Very likely to improve your symptoms
	Might improve your symptoms*
Modality	Home visit
	Phone
	Online
	Clinic visit*
Availability of childcare	Free childcare available
	No childcare available*

*Base level

^+^Recommended in evidence-based clinical practice guidelines

Online, telephone-based and home visiting modalities plus the provision of free childcare (postulated to reduce inconvenience barriers) were incorporated to explore future possibilities. During preliminary testing, some respondents found a few alternatives involving online or telephone-based modalities unrealistic, so additional information was added on how these could function in practice.

Treatment types included those provided in public and private sectors, within and outside traditional healthcare services, to explore women’s preferences over the range actually utilised. Table 1 indicates which are recommended in evidence-based guidelines [18, 19, 21].

The efficacy attribute (described as “chance of improving symptoms” in the survey) permitted separate exploration of preferences for treatment type and stated efficacy. In preliminary testing respondents reported that they found a quantitative attribute unfeasible; the levels “very likely to improve your symptoms” or “might improve your symptoms” were thought to better reflect advice given in healthcare settings.

Cost was included to explore its strength as a barrier to treatment. The survey specified up-front cost, not accounting for rebates or refunds women might expect from public or private health insurance, as this was thought to be a stronger barrier than net out-of-pocket cost. We also asked whether participants held private health insurance that covered non-hospital expenses.

### 2.2 Experimental design

The software Ngene (v1.1.1) was used to produce a fractional factorial orthogonal main effects design. For attribute level balance the number of choice sets was a multiple of eight (as treatment type had eight levels), and to ensure sufficient degrees of freedom for the number of parameters to be estimated, the minimum number of choice sets was sixteen. The design contained thirty-two choice sets, which were blocked into two versions to reduce cognitive burden. Completing sixteen choice sets has been found to have little effect on participants’ self-assessed certainty, perceived level of difficulty or variability of responses [50].

### 2.3 Recruitment of participants

Participants were women in the perinatal period, representing the population at risk of PNDA and therefore potential consumers of PNDA treatment services. This avoided missing the preferences of women who were not identified as experiencing or at risk of PNDA due to inadequate screening, avoidance due to stigmatisation or other similar reasons. Limiting the sample to women in the perinatal period facilitated placing themselves in the hypothetical choice context. Participants were recruited within Australia through an online research company, which has a panel of potential survey participants and can expedite access to people of the appropriate age and gender, in this case perinatal women. Women in the panel aged 18–45 were sent a request to participate in the survey by the company on behalf of the study team, and the sample was further limited, through screening questions, to women who were either currently pregnant or had given birth in the previous 12 months. The characteristics of the sample were compared to Australian national data using Pearson’s chi-square tests, to explore their representativeness of the population of perinatal women.

Formal sample size calculations for DCEs require prior estimates of the attribute parameter values under investigation, which are not known before undertaking the research. Therefore DCEs have traditionally used rules of thumb when determining sample size [51]. The approach proposed by Orme [52], based on the highest number of levels in any attribute in the study, the number of choice sets and the number of alternatives, would estimate a minimum sample size of 125 participants for this study.

### 2.4 Data collection

**Fig 1:**
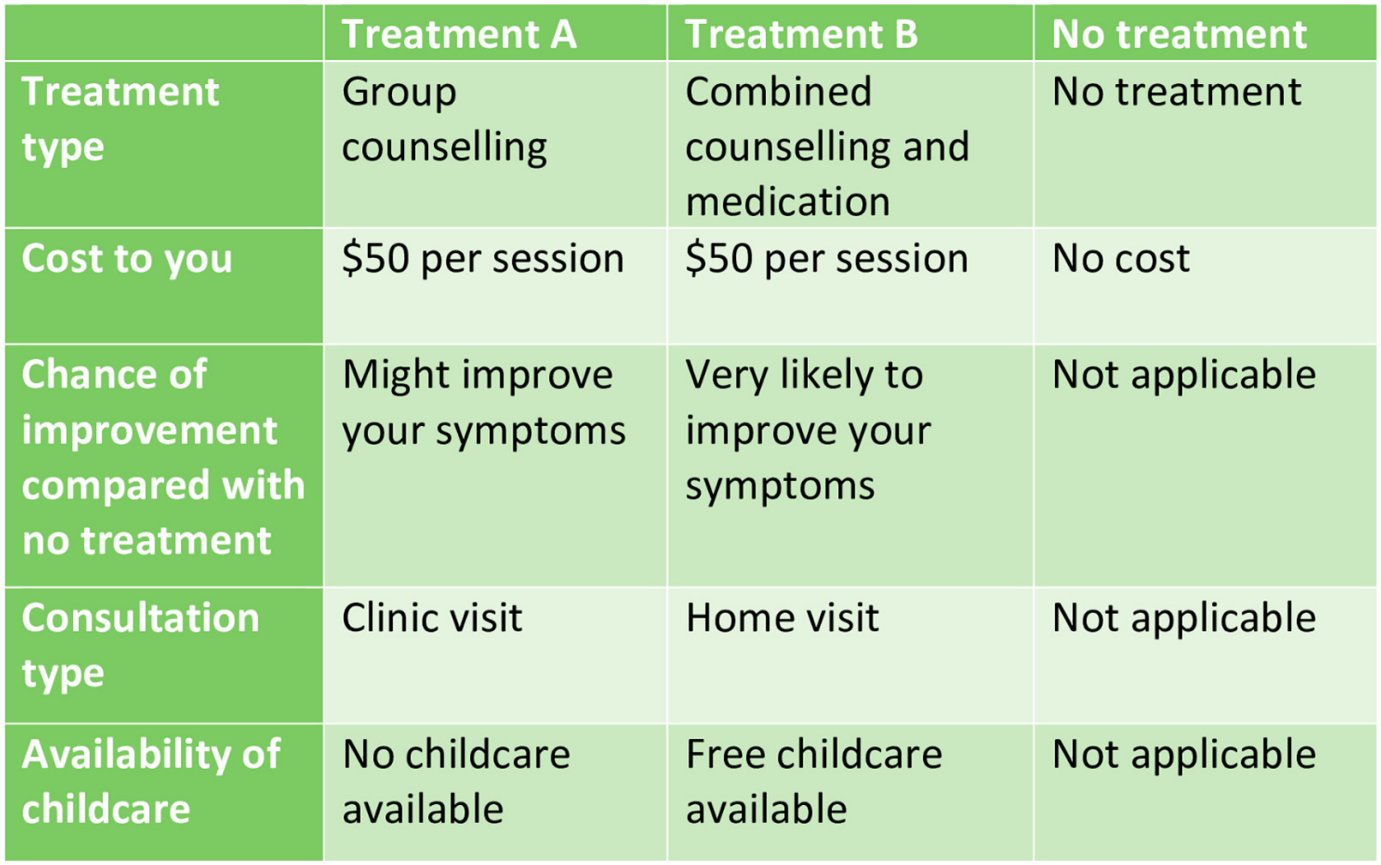
Sample choice set.

The study was approved by the Monash Health Human Research Ethics Committee and the Monash University Human Research Ethics Committee. The choice sets were administered in an anonymous online survey, which began by providing information about participating in the study. Respondents were asked to provide implied consent by proceeding through the survey after the information screen, following which they were provided with information on PNDA and descriptions of each attribute level to promote consistency in their understanding of the terms. Participants completed sixteen choice sets, each of which consisted of the two treatment alternatives and the “No treatment” alternative described according to the five attributes in the format as in Fig 1, with a question below asking the participant to select their most preferred option. At the end of the survey participants completed questions about their socio-demographic characteristics, with the range of questions informed by those characteristics which have been found to affect attitudes to mental health treatment [53–55] or were hypothesised to influence treatment uptake.

To set up the choice context, participants were asked to imagine that they had symptoms of PNDA (as described in the first part of the survey), and that their midwife, GP or maternal and child health nurse had identified this. They were asked to nominate the alternative in each choice set that they would choose if offered those treatment options in ‘real life’.

### 2.5 Analysis

From participants’ recorded choices we estimated an indirect utility function, which summarises the expected utility (an unobserved entity) for the individual derived from combinations of attributes (which are observed). Choice models assume that an individual will choose an alternative if and only if she expects to derive higher utility from it than from other available alternatives. Under random utility theory, utility is conceptualised as deriving from observed (*V)* and unobserved (*ε*) components [56]. We specified a utility function that allowed us to explore both the women’s preferences for treatment attributes and how their preferences differed according to sociodemographic characteristics. If *V* is expressed as attributes of alternatives (*X*) and characteristics of choosers (*Z*), the utility function for individual *i* facing alternative *j* in choice set *s* can be expressed as

$$U_{ijs}=X_{ijs}\prime \beta_{j}+Z_{i}\prime \gamma +\varepsilon_{ijs}$$ (1)

We first estimated a conditional logit model. However, we also implemented more flexible models to relax some of its restrictive assumptions, improve goodness of fit and allow a theoretically richer interpretation of the observed choice heterogeneity. The conditional logit model accommodates observed heterogeneity, from the inclusion of characteristics as *Z* variables, but not unobserved heterogeneity deriving from differing tastes among individuals with the same characteristics, or from differences in the randomness of choice. It is limited to proportionate substitution patterns arising from the independence of irrelevant alternatives property, and does not account for correlations in unobserved factors across choice sets completed by the same individual [56].

The mixed logit model overcomes these limitations, except that it does not address variation in randomness of choice (termed scale heterogeneity) [56]. In the mixed logit model we specified lognormal distributions for the cost and efficacy parameters to fit *a priori* understanding of their potential range (negative and positive respectively, with cost transformed to its negative) and allowed other attribute coefficients to derive from normal distributions. Restricting parameter distributions, when the expected sign is known, has been shown to improve goodness of fit compared with allowing a normal distribution for all parameters [57].

In conditional logit and mixed logit models the estimated coefficients are actually an expression of the parameters multiplied by the scale parameter, *σ*, which is inversely related to the variance of the error term [56]. Assuming that all variation in this confounded term arises from unobserved preference heterogeneity (setting *σ* to equal one) implies that all individuals have the same degree of randomness in their choice. We explored models that attempt to capture scale heterogeneity, including a heteroskedastic conditional logit model, in which scale varies as a function of specified observed characteristics of the chooser, but no separate preference heterogeneity is accommodated. We also estimated a generalised multinomial logit model [58], which allows unobserved preference heterogeneity, via estimation of random parameters, and scale heterogeneity, acknowledging that this may be interpreted as permitting a more flexible distribution for confounded scale and preference heterogeneity, rather than separately estimating scale [59]. Further information on the estimated models can be found in S2 Text.

Each model included an alternative-specific constant (ASC) for treatment. The ASC represents a composite of participants’ underlying preference for treatment over non-treatment (any unobserved features of treatment not described by the attributes) along with preferences for the attributes’ base levels. Sociodemographic characteristics entered as interactions with either the ASC or treatment attributes. Where there was no *a priori* reason to apply one specification (e.g. help-seeking intention relating to preference for treatment over non-treatment, or private health insurance moderating the effect of cost), we undertook an iterative process examining the variable’s statistical significance as a predictor of choice under each specification.

Whilst the sign and statistical significance of the estimated coefficients are meaningful, to evaluate the relative impact of each attribute on choice we utilised predicted probability analysis [60], as commonly used in DCEs in health (e.g. [61, 62]). Setting the attributes to their base levels, the predicted probability of choosing treatment versus non-treatment is estimated for each individual in the sample, and the same for an alternate treatment package. Systematically varying each attribute over its levels in the alternate package, we produced a ranking of the importance of each attribute to choice. The base attribute levels were: zero cost, clinic visit, medication, no childcare and efficacy described as “might improve symptoms”.

To evaluate the impact of potential policy changes on treatment uptake we estimated the mean predicted probability with which specified treatment types and non-treatment would be chosen, with levels of the attributes under policy-change scenarios informed by real-world services and guideline recommendations.

We present the predicted effects of two policies suggested by the estimation results, including the impacts on uptake of each treatment type, proportions choosing guideline-recommended and non-recommended treatments and non-treatment, and how these effects differ by women’s sociodemographic characteristics. Additionally, we describe the treatment packages predicted to achieve the highest uptake of recommended treatment for different types of women, and explore the uptake of non-recommended treatments in direct comparisons with two recommended treatment types (medication versus individual counselling).

## 3. Results

### 3.1 Participants

In total, 1,264 women referred by the online research company were excluded at the screening stage because they were not in the perinatal period, a further 354 dropped out during the survey and a total of 217 women who completed the full survey formed the final sample. No further information is available on those who dropped out. The final sample (who completed the survey in April 2014) had similar sociodemographic characteristics to national data for perinatal women, as shown in Table 2. This sample size exceeded the usual rules of thumb for DCE sample size estimation, including that derived (N = 125) using the approach suggested by Orme [52). Most participants were either pregnant (42%) or breastfeeding (40%); 20% were neither and 3% were both. Most (81%) had more than high school education. Over half (57%) had experienced at least one of the treatment types in the study; the remaining 43% stated that they had not experienced any of the treatment types. Approximately three-quarters stated that they would seek professional help if experiencing symptoms of PNDA, similar to help-seeking rates in observational studies [63].

**Table 2:** Summary of participant characteristics.

Characteristic	Sample (n = 217)	National data^a^
Age (years)		
Mean	32	30^b^
Highest educational attainment (%)		
Year 12 (completion of high school) or less	19	23^c^
Diploma, certificate, bachelor or postgrad.	81	77^c^
Annual household income (%)		
≤$25999	4	12^c^
$26000–51999	16	17^c^
$52000–88399	33	28^c^
$88400–155999	39	32^c^
$156000+	8	11^c^
Country of birth (%)		
Australia	71	70^b^
Language spoken at home (%)		
English	86	89^c^
Area of residence (%)		
Major city	75	71^b^
Inner/ outer regional	24	26^b^
Remote/ very remote	1	3^b^
Marital status (%)		
Married or living with partner	93	93^c^
Private health insurance (%)		
Holds insurance that covers non-hospital expenses	59	
Currently pregnant (%)	42	
Currently breastfeeding (%)	40	
Number of children living with her (%)		
0	13	
1	48	
2–3	33	
4 or more	6	
Employment status (%)		
Home duties or paid maternity leave	54	
Full or part time paid	40	
Unemployed, student or unable to work	6	
Past history of treatment types (not limited to the perinatal context)		
Medication (antidepressant, anti-anxiety or antipsychotic)	26	
Individual counselling	32	
Group counselling	8	
Natural, herbal or traditional Chinese medicine	16	
Early parenting centre programme	13	
Meditation, yoga or exercise	22	
Peer support	5	
None	43	
Self-perceived level of support		
A lot	66	
Some	31	
None	3	
Stated help seeking		
Would seek help if had symptoms of PNDA	77	

^a^:Blank indicates data not available for national perinatal population

^b^:Source: Li Z, Zeki R, Hiilder L, Sullivan EA. Australia's mothers and babies 2011. Canberra: Australian Institute of Health and Welfare; 2013.

^c^:Source: AIHW. Perinatal depression: data from the 2010 Australian National Infant Feeding Survey. Canberra: Australian Institute of Health and Welfare; 2012.

### 3.2 Estimation results

After comparison of the estimated models, mixed logit was preferred as it demonstrated not only the best fit to the data (summarised by the AIC and BIC), but overcame most limitations of CL and accommodated lognormal random parameter distributions. It might have been expected that the generalised multinomial logit, which has the added advantage of allowing for scale heterogeneity as well as random parameters, would be the preferred model. However, the estimation software (Stata v13) could not accommodate non-normal random parameter distributions in generalised multinomial logit, which may explain the better fit of mixed logit in this case [57]. Table 3 presents the results of conditional logit (the basic model for comparison) and mixed logit (the preferred model). The table first presents the ASC, representing the preference for treatment over non-treatment that is not explained by the attributes, followed by the attribute level parameters in the order in which they were presented to participants. The latter part of Table 3 presents the sociodemographic characteristics included in the model, interacted with either the ASC or a treatment attribute (in order to vary over the choice sets and therefore remain in the model for estimation). As heteroskedastic conditional logit and generalised multinomial logit models are not used in further analysis, we present those results, which demonstrate significant unobserved scale heterogeneity and significance of marital status and number of children for parameterised scale heterogeneity, in S1 Table.

**Table 3:** Model estimates.

	Conditional logit		Mixed logit			
		*s*.*e*.	Mean	*s*.*e*.	Std. dev.^†^	*s*.*e*.
Treatment vs. non-treatment (alternative-specific constant)	1.696***	(0.381)	3.337	(1.709)	2.883***	(0.300)
**Attributes of treatment services**						
Treatment types						
Counselling	0.965***	(0.216)	1.198***	(0.330)	-0.334	(0.244)
Counselling & medication	1.466***	(0.208)	1.749***	(0.340)	0.901***	(0.236)
Peer support	0.681**	(0.225)	0.855**	(0.327)	-0.542*	(0.273)
Group counselling	0.696***	(0.205)	0.743*	(0.319)	0.502	(0.257)
Early parenting centre programme	1.331***	(0.196)	1.198***	(0.335)	-0.941***	(0.232)
Natural, herbal or traditional Chinese medicine	0.983***	(0.207)	1.094**	(0.363)	1.205***	(0.215)
Meditation, yoga or exercise	1.452***	(0.208)	1.601***	(0.343)	0.845***	(0.211)
Medication	*base level*					
Cost	-0.0148***	(0.00110)	-0.0562***	(0.00769)	0.125***	(0.0351)
Efficacy						
Very likely to improve	0.387***	(0.0573)	0.797***	(0.133)	2.187***	(0.830)
Might improve	*base level*					
Modalities						
Home visit	-0.0589	(0.0849)	0.131	(0.109)	-0.0253	(0.190)
Telephone	-0.220**	(0.0818)	-0.216	(0.114)	-0.250	(0.154)
Online	-0.0851	(0.0815)	0.0467	(0.121)	-0.153	(0.403)
Clinic visit	*base level*					
Childcare						
Free childcare available	0.223***	(0.0568)	0.224**	(0.0860)	0.451***	(0.136)
No childcare available	*base level*					
**Sociodemographic characteristics interacted with alternative-specific constant**						
Age	-0.0359***	(0.0112)	-0.0548	(0.0476)		
In paid employment	0.229*	(0.112)	-0.257	(0.492)		
Unemployed, student or unable to work	-1.378***	(0.177)	-2.515***	(0.742)		
Experience of any treatment type/s	0.614***	(0.109)	1.152*	(0.524)		
Lower support levels	-0.637***	(0.103)	-0.869	(0.477)		
States would seek help	0.333**	(0.117)	1.113	(0.610)		
**Sociodemographic characteristics interacted with attributes of treatment services**						
Income X Cost	-3.95x10^-6^	1.09x10^-5^	-1.21x10^-5^	1.94x10^-5^		
Private health insurance X Cost	0.00328***	(0.000892)	0.00408*	(0.00205)		
Experience of matched treatment type	0.351***	(0.0850)	0.606***	(0.133)		
Education up to high school interacted with treatment type						
Counselling	-0.596*	(0.233)	-0.251	(0.399)		
Counselling & medication	-0.724***	(0.226)	-0.402	(0.393)		
Peer support	-0.206	(0.242)	-0.256	(0.386)		
Group counselling	-0.131	(0.217)	-0.337	(0.388)		
Early parenting centre programme	-0.0493	(0.218)	-0.906*	(0.426)		
Natural, herbal or traditional Chinese medicine	-0.175	(0.218)	-0.454	(0.444)		
Meditation, yoga or exercise	-0.715**	(0.228)	-0.232	(0.413)		
Breastfeeding interacted with treatment type						
Counselling	-0.102	(0.227)	-0.00132	(0.375)		
Counselling & medication	-0.774***	(0.221)	-0.800*	(0.380)		
Peer support	-0.152	(0.238)	-0.0727	(0.365)		
Group counselling	-0.129	(0.218)	-0.194	(0.365)		
Early parenting centre programme	-0.531*	(0.214)	-0.347	(0.378))		
Natural, herbal or traditional Chinese medicine	-0.520*	(0.217)	-0.427	(0.411)		
Meditation, yoga or exercise	-0.277	(0.222)	-0.00732	(0.387)		
Pregnant interacted with treatment type						
Counselling	-0.201	(0.228)	-0.0593	(0.374)		
Counselling & medication	-0.824***	(0.221)	-0.981**	(0.378)		
Peer support	-0.527*	(0.239)	-0.477	(0.362)		
Group counselling	-0.392	(0.215)	-0.324	(0.360)		
Early parenting centre programme	-0.405	(0.212)	-0.268	(0.377)		
Natural, herbal or traditional Chinese medicine	-0.448*	(0.217)	-0.343	(0.411)		
Meditation, yoga or exercise	-0.482*	(0.220)	-0.463	(0.387)		
*AIC*	5758.1		4660.0			
*BIC*	6077.1		5080.6			
LL	-2835.1		-2272.0			

Standard errors in parentheses

*p<0.05,

**p<0.01,

***p<0.001

^†^The sign of the estimated standard deviations is irrelevant and can be interpreted as being positive

Examining the mixed logit results, all attributes except modality were significant predictors of choice. As expected, lower cost, higher efficacy and childcare increased the likelihood that a treatment package would be chosen. All treatment types were significantly preferred to medication. Whilst no level of the modality attribute reached the 5% level of statistical significance, telephone-based provision was negative and of borderline significance (p = 0.06). All attributes with random parameters exhibited significant preference heterogeneity (evidenced by significant standard deviations) except for one treatment type (counselling) and the modality variables, highlighting an important limitation in the CL model, which assumes homogeneous preferences.

Women’s perinatal phase (whether she was pregnant, postnatal and breastfeeding or postnatal but not breastfeeding) and level of education (whether or not she had more than high school education, a natural cut-off point) were associated with significant differences in preferences for treatment type, and private health insurance moderated the effect of higher costs. Compared with non-breastfeeding postnatal women, pregnant and breastfeeding women had lower preference for the combination of medication and counselling. Having no more than high school education was associated with higher preferences for early parenting centre programmes.

Past experience of any of the treatment types increased preference for treatment overall, and past experience of the treatment type offered in a given scenario increased the likelihood of uptake further still. Being unemployed, a student or unable to work (compared with maternity leave or home duties) decreased treatment uptake. Treatment uptake was lower among women who perceived that their level of support was “some” or “none” (rather than “a lot”), and higher among women who stated that they would seek help if they had PNDA.

### 3.3 Relative importance of attributes

**Fig 2:**
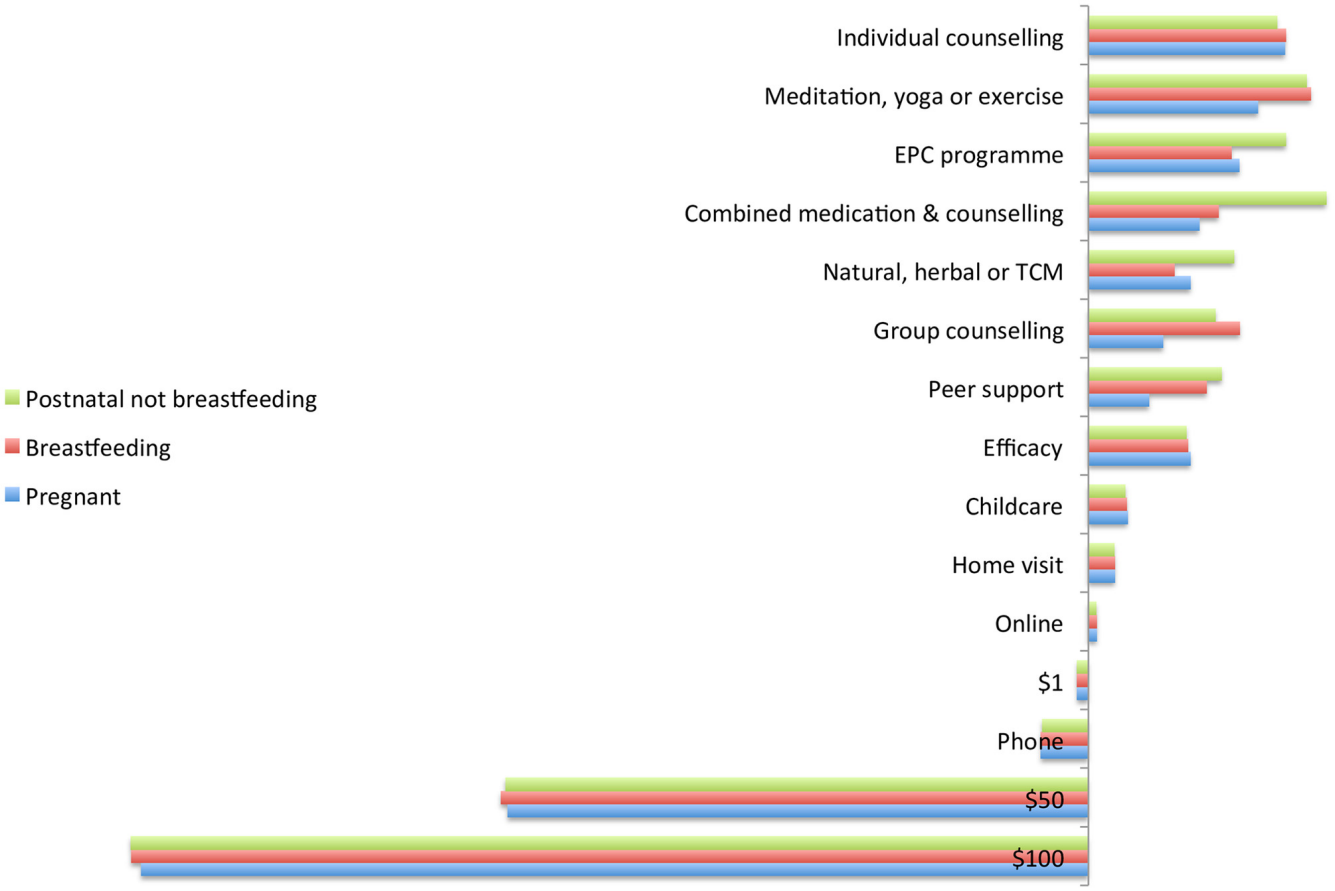
Relative importance of attributes by perinatal phase.

**Fig 3:**
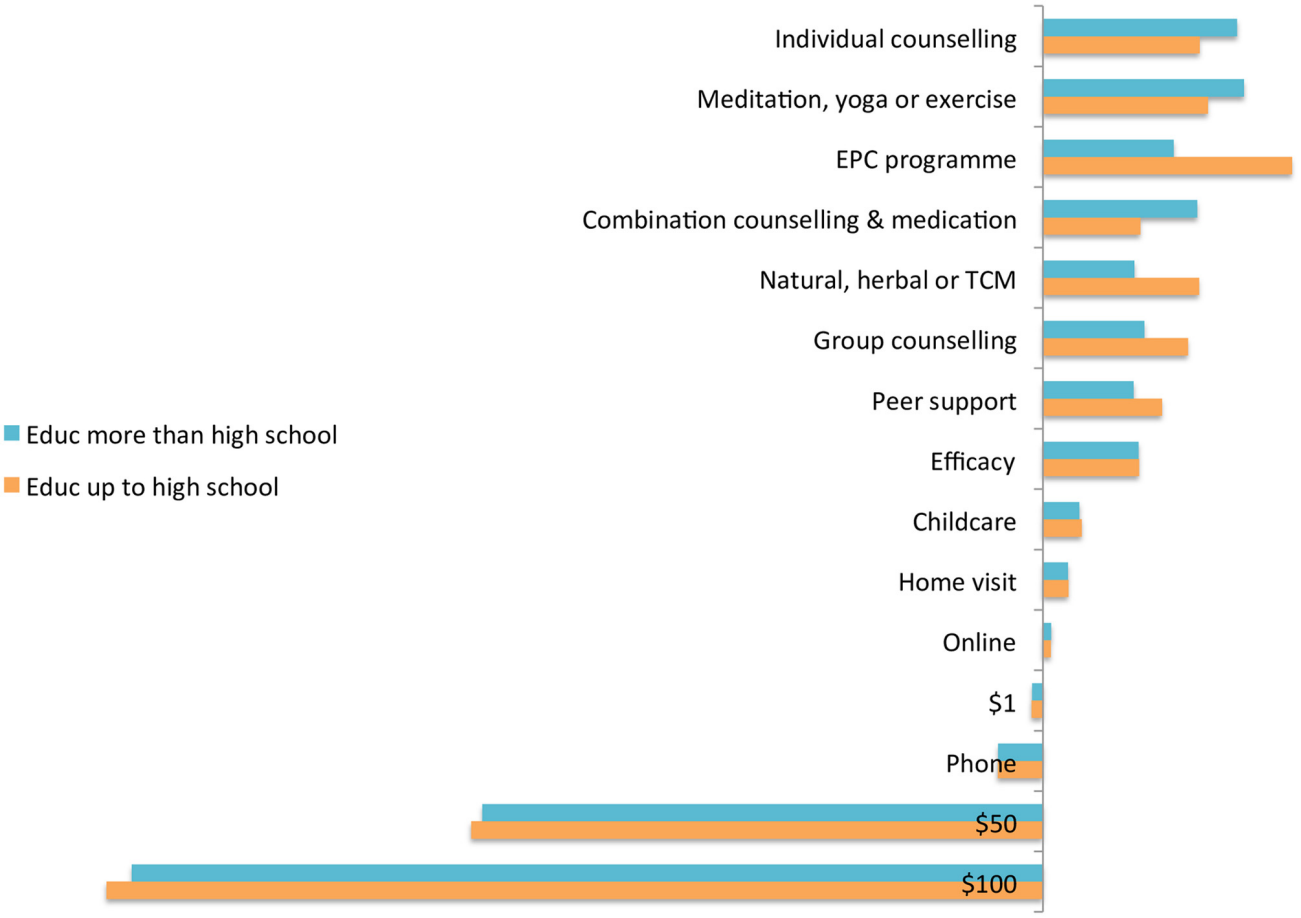
Relative importance of attributes by highest educational attainment.

Figs 2 and 3 present the results of predicted probability analysis in which each attribute was systematically varied from the base level, separately by women’s perinatal phase and education level. Cost (over AU$50) and treatment type consistently had the greatest impact. The preference against medication is in keeping with previous findings [29]. The impact of cost also confirms that this is a substantial barrier to treatment. Stated efficacy had lower impact than treatment type or cost, whilst provision of free childcare had even less. Consistent with its non-significance as a predictor of choice, modality had little impact.

The ranking of treatment types varied with women’s perinatal phase and education. The highest ranked treatment type for pregnant women was individual counselling, for breastfeeding women was meditation, yoga or exercise and for non-breastfeeding postnatal women was combined counselling and medication. The highest ranked for women with lower levels of education was early parenting centre programmes, and for women with higher levels of education was meditation, yoga or exercise. However, individual counselling was consistently the highest ranked guideline-recommended treatment, apart from non-breastfeeding postnatal women, who ranked counselling combined with medication higher.

### 3.4 Policy analysis: Predicted uptake

Attribute levels in the base case for policy analysis were derived from real-world services and guideline recommendations, assuming no reimbursements by public or private health insurance, clinic visits and no childcare. Table 4 details the costs of treatment packages under base case and policy change scenarios and the sources of these estimates. Across all eight treatment types, predicted uptake was 93%, higher than that found in observational studies. Unobserved factors may differ between the two situations [56]; for example stigma and inconvenience may have had smaller impact in the DCE. We adjusted for these differences by recalibrating the ASC, which captures the average effect of unobserved factors [56]. We recalibrated to predicted treatment uptake of 60% (approximating best available estimates of observed uptake) for the base case. Since women in observational studies may have chosen from among a narrower range than our scenario of eight treatment types, these recalibrated estimates are likely to be conservative. As shown in Table 5, this scenario predicts only 27% of women to take up guideline-recommended treatments, 33% non-recommended treatments and 40% forgoing treatment altogether. Early parenting centre programmes have the highest predicted uptake, followed by peer support.

**Table 4:** Cost of treatment types for base case and subsidies policy.

Treatment type	Base case	Subsidies policy
Individual counselling	$235^a^	None^e^
Group counselling	$47^a^	None^e^
Medication	$75.65^b^	$6.10^e^^,^^f^
Meditation, yoga or exercise	$25^c^	$25^c^
Natural, herbal or traditional Chinese medicine	$30^c^	$30^c^
Early parenting centre programme	None^d^	None^d^
Combined medication & counselling	$310.65^a^^,^^b^	$6.10^e^^,^^f^
Peer support	$10^c^	None^e^

^a^Based on Australian Psychological Society recommended rates for 46–60 minute consults

^b^Based on Australian Medical Association recommended level B consult rate (minus Medicare rebate) plus Pharmaceutical Benefits Scheme fee for general patients

^c^Based on market rates

^d^Based on bulk-billing or other government subsidisation of most services at early parenting centres

^e^Assumes full universal bulk billing of guideline-recommended treatments for PNDA

^f^Based on Pharmaceutical Benefits Scheme fee for concessional patients

**Table 5:** Predicted % uptake under base case and policy change scenarios*.

	Guideline-recommended treatment^+^					Non-recommended treatment			No treatment	
Scenario	Individual counselling	Medication	Medication & counselling	Group counselling	Peer support	EPC^†^	MYE^#^	Natural, herbal or TCM^		Total
Base case^1^	2.61	2.11	2.29	6.20	13.30	19.35	11.12	3.02		
				*Subtotal* 26.51			*Subtotal* 33.49		40.00	100
Subsidies policy^2^	16.08	3.71	13.57	9.97	9.03	10.82	6.92	2.28		
				*Subtotal* 52.36			*Subtotal* 20.02		27.62	100
Childcare added to subsidies^3^	17.31	4.01	14.51	10.75	9.75	9.77	6.24	2.07		
				*Subtotal* 56.33			*Subtotal* 18.08		25.59	100

*See Equation 5 in S2 Text for method of calculation. Uses recalibrated values so that in the base case predicted uptake was 60%, which approximates best available estimates of observed treatment uptake.

^+^Recommended in evidence-based clinical practice guidelines [18, 19, 21]

^†^EPC = Early parenting centre programme

^#^MYE = Meditation, yoga or exercise

^TCM = Traditional Chinese medicine

^1^ Base case: Cost from column 1 of Table 4, clinic visit, no childcare

^2^ Subsidies policy: Cost from column 2 of Table 4, clinic visit, no childcare

^3^ Childcare added: Cost from column 2 of Table 4, clinic visit, free childcare available

Guided by the importance of the cost attribute, we estimated the impact of subsidising recommended treatments. As seen in Table 5, compared to the recalibrated base case, non-treatment reduces from 40% to 28%, recommended treatment doubles (from 27% to 52%) and non-recommended treatment drops from 33% to 20%. The largest increases are for individual counselling and combined counselling and medication; still less than 4% are predicted to choose medication alone.

Consistent with the lower relative importance of the childcare attribute, providing free childcare with recommended treatments had a much smaller estimated impact than the subsidies policy, with a four-percentage-point increase in recommended treatments and two-percentage-point reductions in non-treatment and non-recommended treatment.

**Fig 4:**
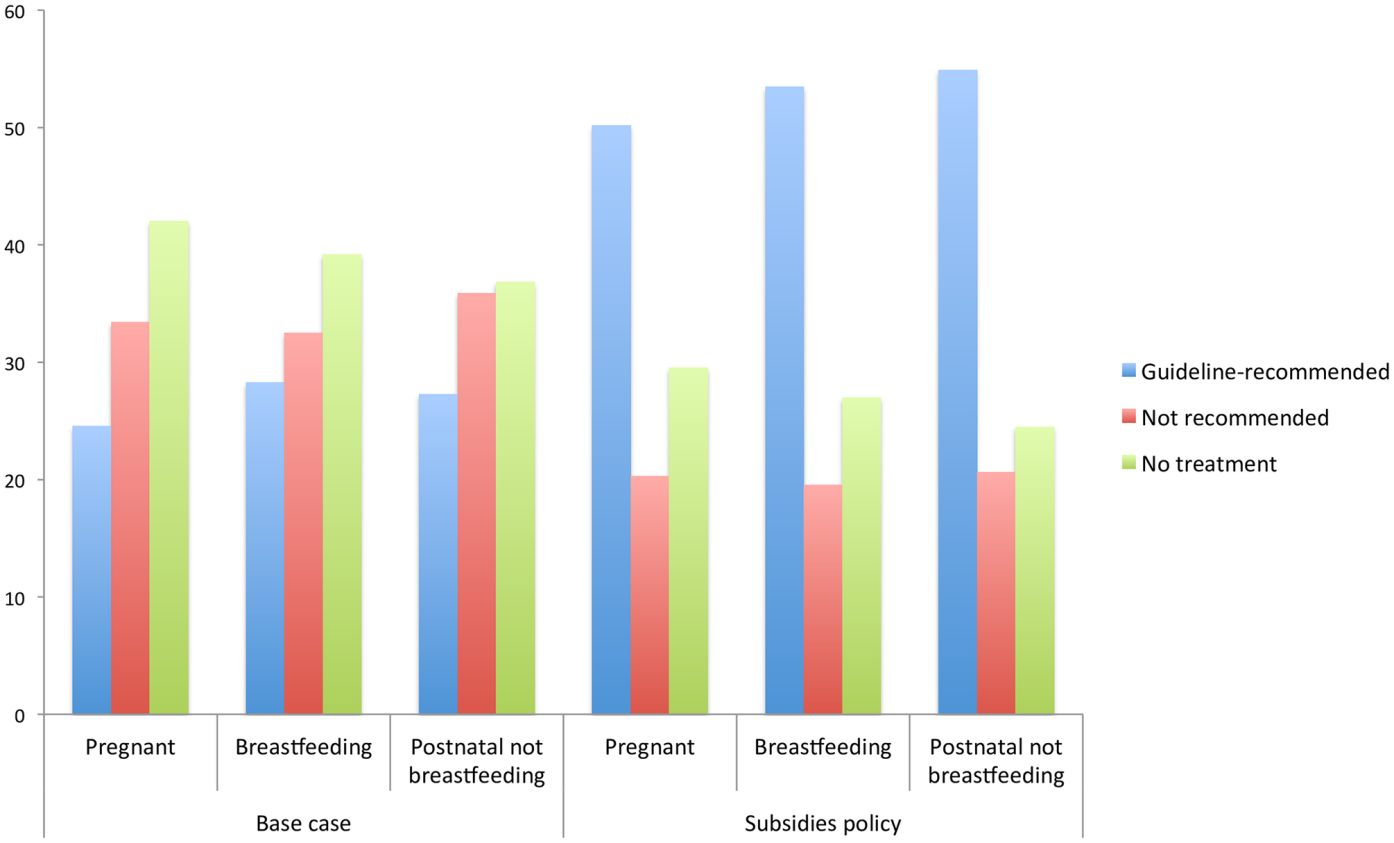
Rates of treatment (guideline-recommended and non-recommended) and non-treatment by perinatal phase (base case and subsidies).

Predicted uptake differs (as shown in Fig 4) by perinatal phase: non-treatment among non-breastfeeding postnatal women is less than that among either pregnant or breastfeeding women. Under the base case, this is largely driven by higher uptake of early parenting centre programmes and peer support among the non-breastfeeding postnatal group, and under the subsidies policy, by higher uptake of combined counselling and medication among them.

**Fig 5:**
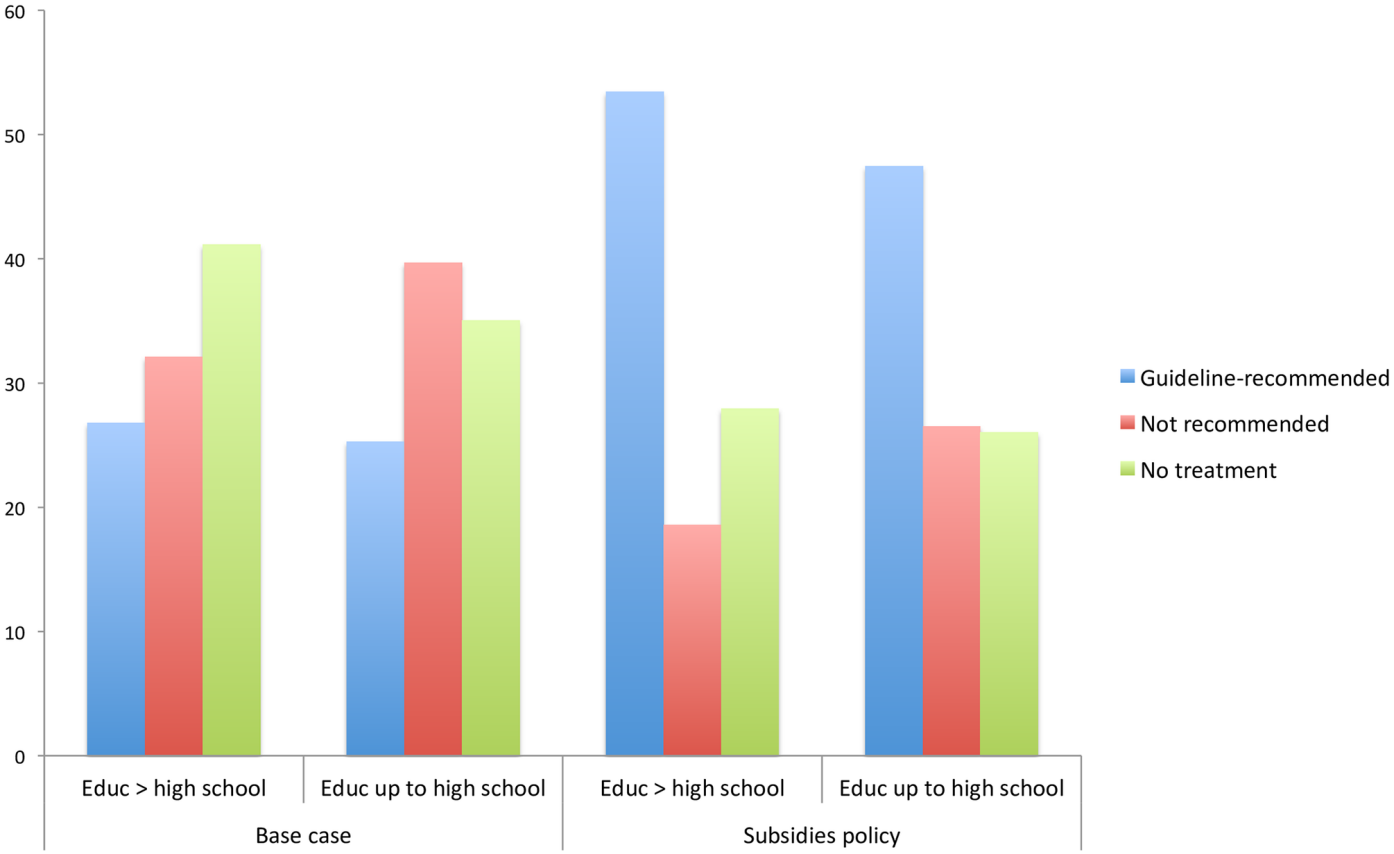
Rates of treatment (guideline-recommended and non-recommended) and non-treatment by highest educational attainment (base case and subsidies).

Looking at differences according to educational attainment, as shown in Fig 5, although the subsidies policy increases predicted uptake of recommended treatment in both groups by more than 20 percentage-points, the policy widens the gap between those with higher and lower levels of education.

Predicting the uptake of a single, optimal treatment package for each subgroup by perinatal phase and educational attainment also demonstrates the importance of women’s own characteristics. For each group we estimate uptake of the preferred guideline-recommended treatment type (individual counselling for most groups, except combined counselling and medication for non-breastfeeding postnatal women) at no cost, in home visits, with childcare provided. This achieves four to nine percentage point higher uptake among non-breastfeeding postnatal women and those with higher education.

To explore why women would choose a treatment with lower likelihood of effectiveness, we compared uptake of a non-recommended treatment and a recommended treatment, both at no cost and applying their real-world efficacy. When comparing meditation, yoga or exercise with medication, the model predicts 19% of women would choose the recommended treatment, and 49% no treatment. Comparing counselling (instead of medication) with meditation, yoga or exercise, recommended treatment rises to 35% and non-treatment reduces to 41%. A similar pattern arises when comparing natural, herbal or traditional Chinese medicine with either medication or counselling.

## 4. Discussion

This study, the first DCE of women’s preferences for treatment of PNDA, provides not only further insight into preferred attributes of treatment for PNDA, but quantifies the relative importance of those attributes to women’s choices, the influence of women’s own characteristics on their choices, and presents predictions of policy impact.

### 4.1 Policy and practice implications

These results provide supporting evidence for measures to reduce the cost barrier of recommended treatments, particularly individual-based psychological therapies, to substantially reduce both non-treatment and women choosing non-recommended treatments. Combined counselling and medication was most acceptable to non-breastfeeding postnatal women, but also faces a cost barrier. Group-based counselling may facilitate access to effective treatment at lower per-person cost, being preferred to medication but not to individual counselling. Preference for individual over group counselling is hypothesised to arise from stigma [55], which may also drive the lower preference for peer support. Although women have readily used informal peer support for perinatal depression [30] it is possible they did not view this as “treatment”.

The lower impact of stated efficacy compared with treatment type might relate to its qualitative nature, which reflected the uncertainty faced in applying the efficacy evidence to an individual woman. However, women do (in ‘real life’) choose non-evidence-based treatments. Rather than an irrational choice of something they believe to be ineffective, this may reveal lack of knowledge regarding treatment efficacy, or prioritisation of their own ideas of efficacy over the evidence; either interpretation reinforcing the need to educate women about treatments. It may also reflect trade-offs between efficacy (health outcomes) and the process of getting well [64]. If health outcomes were the only thing that mattered to women (and if they considered the efficacy attribute to accurately reflect health outcomes), no other attribute would be of significance in the utility function. Focus group participants discussed the importance of the quality of relationships with treatment providers (whether they found the person non-judgemental, empathetic and trustworthy), which also emphasised process aspects of treatment.

Higher uptake might be achieved by addressing women’s expectations of treatment. Women may avoid seeking help if they expect only to be offered medication [65]. Our results predict that more women take up non-recommended treatments if they perceive the only effective alternative available is medication (emphasising the importance of understanding non-chosen alternatives). Promoting the availability of other recommended treatments may encourage women to engage with treatment, and not to take up treatments lacking good evidence of efficacy.

Considering individual women’s past experience may be helpful, as treatment was more likely to be taken up in this study if it matched the participant’s previous experience, as others have found [66, 67]. This finding may also suggest merit in targeted education to women without past treatment, whether lack of past treatment indicates no history of depression or anxiety, or a past history but with a negative attitude towards treatment.

These results do not present evidence that providing home visits, telephone consultations or childcare would substantially increase uptake. Some focus group participants commented that they would be reluctant to leave their children with an unknown childcare service, and that having someone come to one’s home could create difficulty for a new mother. However, online consultations, which we found not to reduce uptake compared with clinic visits, offer potential as lower-cost means of delivering treatment than face-to-face visits, especially for psychological therapy [68].

It is noteworthy that even in the best-case scenario some women would still go untreated or choose non-recommended treatments. Uptake was lower among those with less social support and lower levels of education, consistent with framing these as predisposing factors to utilisation [69]. However, the association with education differed across treatment types and may operate by reducing women’s perceptions of stigma and barriers to PNDA treatment [54]. Importantly, lower education was associated with lower preferences for cornerstones of PNDA treatment, counselling and medication. Pregnancy and breastfeeding reduced the likelihood of uptake, which may relate in part to concerns about the baby’s safety, particularly in relation to medication [30]. Educating women about the risks of untreated PNDA may help overcome this [21, 29]. However, the lower preference for treatment associated with pregnancy and breastfeeding was not limited to treatments involving ingestion of a medicinal substance; other factors such as time pressures or focussing on the baby’s wellbeing rather than the mother’s may contribute.

### 4.2 Limitations and future research possibilities

The requirements that participants used a computer with Internet access and were registered with the online research company may have contributed the finding that online modality was comparable with clinic visits.

Participants for this study were recruited in an Australian context, which might limit its applicability in other countries. Future research in other countries could compare women’s preferences across differing populations, cultures, health service arrangements and other contextual factors.

Participants were in the perinatal period, and although by chance some would have been experiencing PNDA, and many barriers to uptake relate to the perinatal period rather than depression or anxiety itself, a similar study involving only women with PNDA may provide additional evidence in this field. For this study, whilst we would have preferred to ask about participants’ current and past mental health history, we did not receive ethical approval to include such questions in the anonymous online survey. However, recruiting women based on their PNDA status would risk excluding those who had PNDA but had avoided or missed out on being identified as such. The preferences of these women are of key importance to the question of improving treatment uptake. It is also important to include women without experience of the treatments, as we did. This group may be a target for policy intervention since they have lower uptake of treatment, especially considering that for many women PNDA may be their first encounter with such treatments.

As in the majority of health-related DCEs [42], this study used a main effects design, which assumes non-significance of attribute interactions and an additive indirect utility function. DCE guidelines recommend inclusion of interactions, where possible, to relax these assumptions [41, 70, 71]. While main effects can have “reasonable predictive accuracy even when utility functions are quite misspecified”, for practical, policy-focussed research objectives such as ours [71, p.89], including attribute interactions in future studies would permit exploration of such effects.

Although DCEs elicit preferences in the context of a hypothetical market, we endeavoured to provide realistic information in the survey to minimise differences from a real-world situation [72]. In addition, we adjusted for differences in unobserved factors by recalibrating the overall uptake to levels seen in observational studies.

The sample size may limit conclusions that can be drawn. Although the sample size exceeded the rules of thumb usually applied to DCEs and was sufficient to allow estimation of statistically significant coefficients for most treatment attributes, use of a larger sample may have provided more clarity on the impact of modality, since telephone-based provision was of borderline statistical significance in these results.

The treatment type of combined counselling and medication did not specify whether this counselling was individual- or group-based, and so there may have been differing understandings of this treatment type among the participants.

Two potential attributes relating to the treatment provider were excluded at the design stage. Focus group participants’ emphasis on the quality of their relationship with treatment providers was consistent with previous qualitative research [73]. Relationship with the provider was not included in this study as an attribute due to the variation in perception from woman to woman for a particular provider. Provider profession was not included as an attribute since this is usually tied to treatment type. Further research could attempt to disentangle women’s preferences for treatment type, provider type and relationship with provider.

## 5. Conclusion

These results highlight the importance of cost and treatment type to the uptake of treatment for PNDA, and reinforce the need to recognise the likely effects of women’s characteristics on their choices regarding treatment. We have applied DCE methods to consumer preference elicitation in perinatal mental health, an area in which understanding patient preferences may be crucial, given pervasive problems with treatment uptake. Understanding and addressing women’s preferences suggest policy directions for decision makers whose goal is to increase the effective treatment of PNDA and reduce its burden on women, their children and families.

## Supporting Information

S1 Data Data file. (DTA)

S1 Table Heteroskedastic conditional logit and generalised multinomial logit model estimates. (DOCX)

S1 Text Descriptions of attribute levels provided to participants in the survey. (DOCX)

S2 Text Additional details of analysis. (DOCX)
