# Supplementary material for: Women’s Preferences for Treatment of Perinatal Depression and Anxiety: A Discrete Choice Experiment
Source: PLoS One. 2016 Jun 3;11(6):e0156629. doi: 10.1371/journal.pone.0156629 (PMC4892671; doi:10.1371/journal.pone.0156629)
Supplement: S1 Table — (DOCX) [file pone.0156629.s002.docx]

S1 Table. Heteroskedastic conditional logit (CL-HET) and generalised multinomial logit (GMNL) model estimates

|  | CL-HET |  | GMNL |  |  |  |
| --- | --- | --- | --- | --- | --- | --- |
|  |  | *s.e.* | Mean | *s.e.* | Std. dev. | *s.e.* |
| Attributes of treatment services | | | | | | |
| Childcare | 0.101^**^ | (0.0341) | 0.328 | (0.170) | -0.550^***^ | (0.125) |
| Cost | -0.00747^***^ | (0.00150) | -0.0551^***^ | (0.00617) | 0.0411^***^ | (0.00456) |
| Efficacy | 0.192^***^ | (0.0454) | 1.178^***^ | (0.163) | 0.962^***^ | (0.148) |
| Treatment types | | | | | | |
| Counselling | 0.483^***^ | (0.139) | 2.416^***^ | (0.373) | -0.450^*^ | (0.213) |
| Counselling & medication | 0.708^***^ | (0.169) | 1.861^***^ | (0.400) | 2.104^***^ | (0.372) |
| Peer support | 0.340^**^ | (0.128) | 1.405^***^ | (0.350) | -0.215 | (0.176) |
| Group counselling | 0.343^**^ | (0.119) | 1.901^***^ | (0.377) | 0.435 | (0.302) |
| EPC programme | 0.679^***^ | (0.162) | 3.247^***^ | (0.495) | 0.904^***^ | (0.238) |
| Natural, herbal or TCM | 0.476^***^ | (0.138) | 2.019^***^ | (0.333) | 0.993^***^ | (0.199) |
| MYE | 0.705^***^ | (0.168) | 3.166^***^ | (0.501) | 0.814^*^ | (0.346) |
| Modalities | | | | | | |
| Home visit | -0.0226 | (0.0414) | 0.114 | (0.155) | -0.0961 | (0.166) |
| Telephone | -0.111^*^ | (0.0446) | -0.372^*^ | (0.171) | -0.291^**^ | (0.0925) |
| Online | -0.0356 | (0.0401) | 0.00470 | (0.188) | 1.322^***^ | (0.224) |
| Treatment ASC | 0.963^***^ | (0.245) | 3.015 | (1.566) | 3.192^***^ | (0.355) |
| Sociodemographic characteristics interacted with ASC | | | | | | |
| Age | -0.0221^***^ | (0.00646) | -0.0575 | (0.0484) |  |  |
| In paid employment | 0.157^**^ | (0.0586) | 1.108^*^ | (0.502) |  |  |
| Unemployed, student or unable to work | -0.653^***^ | (0.162) | -2.553^*^ | (1.017) |  |  |
| Experience of any treatment type/s | 0.349^***^ | (0.0821) | 1.386^**^ | (0.456) |  |  |
| Lower support levels | -0.291^***^ | (0.0763) | -0.958^*^ | (0.457) |  |  |
| States would seek help | 0.0959 | (0.0667) | 0.888 | (0.497) |  |  |
| Sociodemographic characteristics interacted with attributes of treatment services | | | | | | |
| Income – Cost | 0.00000618 | (0.00000539) | -0.00000730 | (0.0000208) |  |  |
| PHI – Cost | 0.00141^**^ | (0.000518) | 0.00176 | (0.00221) |  |  |
| Experience of matched treatment type | 0.178^***^ | (0.0530) | 0.557^***^ | (0.125) |  |  |
| Education up to high school interacted with treatment type | | | | | | |
| Counselling | -0.299^*^ | (0.130) | 0.0272 | (0.428) |  |  |
| Counselling & medication | -0.363^**^ | (0.129) | -0.260 | (0.433) |  |  |
| Peer support | -0.107 | (0.121) | 0.431 | (0.413) |  |  |
| Group counselling | -0.0904 | (0.109) | 0.376 | (0.408) |  |  |
| EPC programme | 0.0319 | (0.107) | 1.058^**^ | (0.402) |  |  |
| Natural, herbal or TCM | -0.0885 | (0.108) | 0.515 | (0.418) |  |  |
| MYE | -0.363^**^ | (0.132) | -0.0769 | (0.427) |  |  |
| Breastfeeding interacted with treatment type | | | | | | |
| Counselling | -0.0343 | (0.113) | -0.132 | (0.286) |  |  |
| Counselling & medication | -0.361^**^ | (0.129) | 0.0783 | (0.282) |  |  |
| Peer support | -0.0514 | (0.118) | 0.00793 | (0.280) |  |  |
| Group counselling | -0.0250 | (0.109) | -0.0596 | (0.287) |  |  |
| EPC programme | -0.262^*^ | (0.119) | -0.566^*^ | (0.284) |  |  |
| Natural, herbal or TCM | -0.235^*^ | (0.118) | -0.390 | (0.271) |  |  |
| MYE | -0.110 | (0.113) | -0.0677 | (0.321) |  |  |
| Pregnant interacted with treatment type | | | | | | |
| Counselling | -0.117 | (0.114) | -0.525 | (0.303) |  |  |
| Counselling & medication | -0.407^**^ | (0.133) | -0.366 | (0.306) |  |  |
| Peer support | -0.251^*^ | (0.127) | -0.580 | (0.305) |  |  |
| Group counselling | -0.183 | (0.111) | -0.764^*^ | (0.304) |  |  |
| EPC programme | -0.233^*^ | (0.115) | -0.951^**^ | (0.306) |  |  |
| Natural, herbal or TCM | -0.203 | (0.115) | -0.551 | (0.298) |  |  |
| MYE | -0.223 | (0.117) | -0.859^**^ | (0.327) |  |  |
| tau |  |  | 1.276^***^ | (0.106) |  |  |
| *Scale heterogeneity* |  |  |  |  |  |  |
| Country of birth Australia | -0.0303 | (0.0712) |  |  |  |  |
| English spoken at home | 0.180 | (0.100) |  |  |  |  |
| Has spouse or partner | 0.620^***^ | (0.174) |  |  |  |  |
| Number of children | -0.0621^*^ | (0.0268) |  |  |  |  |
| Index of relative disadvantage decile | 0.0142 | (0.0100) |  |  |  |  |
| *AIC* | 5737.3 |  | 4680.0 |  |  |  |
| *BIC* | 6092.6 |  | 5107.8 |  |  |  |
| ll | -2819.7 |  | -2281.0 |  |  |  |

Standard errors in parentheses

^*^ *p* < 0.05, ^**^ *p* < 0.01, ^***^ *p* < 0.001
