# Supplementary material for: Women’s Preferences for Treatment of Perinatal Depression and Anxiety: A Discrete Choice Experiment
Source: PLoS One. 2016 Jun 3;11(6):e0156629. doi: 10.1371/journal.pone.0156629 (PMC4892671; doi:10.1371/journal.pone.0156629)
Supplement: S1 Text — (DOCX) [file pone.0156629.s003.docx]

S1 Text. Descriptions of attribute levels provided to participants in the survey

***Cost to you*** is the amount that you would pay up front for each session of treatment. It does not take into account any rebates from Medicare or private health insurance. In this survey, the cost will range from no cost to $200 per session.

***Chance of improvement*** describes how likely it is that the treatment will improve your symptoms. Every woman is different and so it is not possible to predict an exact chance of recovery.

Each treatment option will be described as:

- Very likely to improve your symptoms, or
- Might improve your symptoms.

***Consultation type*** is how or where you would interact with the person providing your treatment. In this survey the types of consultation will be:

| Clinic visit | You would go to a clinic to see the person providing your treatment. |
| --- | --- |
| Home visit | The person providing the treatment would come to your home to see you. |
| Phone | You would speak to the person providing your treatment over the phone. They could also send you materials for your treatment if needed. |
| Online | You would interact with the person providing your treatment online, which could include chat, email and video calls. If needed, they could also provide materials for your treatment either online or by sending them to you. |

***Availability of childcare*** describes whether free childcare is provided while you receive treatment, or whether no childcare is available. The childcare could be provided at the place you receive treatment, or could be provided elsewhere (including your home) if required.

***Treatment method*** Here is a short description of the different methods:

| One-on-one counselling | Also known as talking therapy. You would attend multiple sessions, usually once per week or fortnight. |
| --- | --- |
| Medication | Antidepressant or anti-anxiety medication, prescribed by a doctor (usually either a GP or a psychiatrist) and taken for several months.  The effects usually take a few weeks to begin.  Possible side effects: nausea, headaches, anxiety, dizziness, weight gain, sexual difficulties, dry mouth and agitation. Sometimes side effects are only short-lived. The medication may pass through to the baby when you are pregnant or breastfeeding so mothers often stop breastfeeding. |
| Combined counselling and medication | You would receive counselling (talking therapy) and take medication.  The possible side effects of medication are the same as if you were taking medication alone, and you would attend multiple sessions with your counsellor. |
| Meditation, yoga or exercise | You would be instructed and supervised in techniques of meditation, yoga or exercise to treat your symptoms of depression or anxiety. |
| Group counselling | You would receive counselling (talking therapy) in a group setting rather than on your own. The other people in the group would also be women experiencing perinatal depression and anxiety. You would attend multiple sessions as for one-on-one counselling. |
| Early parenting centre programme | You would participate in a programme run by an early parenting centre, with a team of nurses, psychologist, social workers, lactation professionals and others.  You and your family would receive support and learn practical skills related to parenting and dealing with depression and anxiety. |
| Natural, herbal or traditional Chinese medicine | Treatments include taking herbs, nutritional supplements or other medicinal substances, acupuncture, massage and cupping. |
| Peer support | You would be introduced to another woman or women who had experienced perinatal depression or anxiety themselves. They would provide emotional support, practical advice and information to help you cope with how you are feeling. |
