# Supplementary material for: Women’s Preferences for Treatment of Perinatal Depression and Anxiety: A Discrete Choice Experiment
Source: PLoS One. 2016 Jun 3;11(6):e0156629. doi: 10.1371/journal.pone.0156629 (PMC4892671; doi:10.1371/journal.pone.0156629)
Supplement: S2 Text — (DOCX) [file pone.0156629.s004.docx]

S2 Text. Additional details of analysis

The mixed logit utility function can be defined as:

$U_{ijs}=X_{ijs}'(\beta+\eta_{i})+\varepsilon_{ijs}$ (2)

The probability that individual *i* will choose alternative *m* is then the integral of the usual logit probabilities weighted by the mixing function $f\left( \beta\right)$:

${Pr}_{ims}=\int\left( {e^{V_{ims}}}/{\sum_{j=1}^{J} e^{V_{ijs}}} \right)f\left( \beta\right)d\beta$ (3)

The mean difference in predicted probability comparing base levels (A) to an alternate package (B) is given by:

$\bar{{\Delta Prob}_{AB}}=\frac{1}{N}\sum_{i=1}^{N} (\hat{{Prob}_{iA}}-\hat{{Prob}_{iB}})$ (4)

The mean predicted probability of a specified treatment package is given by:

$\bar{\mathrm{Prob}_{j}}=\frac{1}{N}\sum_{i=1}^{N} {Prob}_{ji}$ (5)
